# Region-specific mitophagy in nucleus pulposus, annulus fibrosus, and cartilage endplate of intervertebral disc degeneration: mechanisms and therapeutic strategies

**DOI:** 10.3389/fphar.2025.1579507

**Published:** 2025-04-01

**Authors:** Chaoqun Feng, Ziang Hu, Min Zhao, Chuan Leng, Guangye Li, Fei Yang, Xiaohong Fan

**Affiliations:** ^1^ Department of Orthopedics, Hospital of Chengdu University of Traditional Chinese Medicine, Chengdu, China; ^2^ Department of Orthopedics, The TCM Hospital of Longquanyi District, Chengdu, China; ^3^ International Ward (Gynecology), Hospital of Chengdu University of Traditional Chinese Medicine, Chengdu, China

**Keywords:** mechanisms, therapeutic strategies, mitophagy, intervertebral disc degeneration, nucleus pulposus, annulus fibrosus, cartilage endplate

## Abstract

Intervertebral disc degeneration (IVDD) is a prevalent condition contributing to various spinal disorders, posing a significant global health burden. Mitophagy plays a crucial role in maintaining mitochondrial quantity and quality and is closely associated with the onset and progression of IVDD. Well-documented region-specific mitophagy mechanisms in IVDD are guiding the development of therapeutic strategies. In the nucleus pulposus (NP), impaired mitochondria lead to apoptosis, oxidative stress, senescence, extracellular matrix degradation and synthesis, excessive autophagy, inflammation, mitochondrial instability, and pyroptosis, with key regulatory targets including AMPK, PGC-1α, SIRT1, SIRT3, Progerin, p65, Mfn2, FOXO3, NDUFA4L2, SLC39A7, ITGα5/β1, Nrf2, and NLRP3 inflammasome. In the annulus fibrosus (AF), mitochondrial damage induces apoptosis and oxidative stress mediated by PGC-1α, while in the cartilage endplate (CEP), mitochondrial dysfunction similarly triggers apoptosis and oxidative stress. These mechanistic insights highlight therapeutic strategies such as activating Parkin-dependent and Ub-independent mitophagy pathways for NP, enhancing Parkin-dependent mitophagy for AF, and targeting Parkin-mediated mitophagy for CEP. These strategies include the use of natural ingredients, hormonal modulation, gene editing technologies, targeted compounds, and manipulation of related proteins. This review summarizes the mechanisms of mitophagy in different regions of the intervertebral disc and highlights therapeutic approaches using mitophagy modulators to ameliorate IVDD. It discusses the complex mechanisms of mitophagy and underscores its potential as a therapeutic target. The objective is to provide valuable insights and a scientific basis for the development of mitochondrial-targeted drugs for anti-IVDD.

## 1 Introduction

The intervertebral disc, a fibrocartilaginous tissue between adjacent vertebral bodies, consists of the nucleus pulposus (NP), annulus fibrosus (AF) and cartilage endplate (CEP) (Vergroesen et al., 2015). It is a unique structure that provides segmental mobility and is simultaneously responsible for the mechanical stability of the spinal column (Wise et al., 2020). Specifically, the NP resides centrally within the intervertebral disc, fulfilling the role of enduring mechanical impacts (Kepler et al., 2013). Surrounding it intactly, the AF is associated with the regulation of the occurrence of intervertebral disc herniation (Gorth et al., 2020), while the CEP interfaces with the vertebral bones and plays a crucial part in regulating the transport of nutrients (Habib et al., 2023). Residing in a naturally harsh microenvironment of hypoxia, acidic pH, low nutrition and high mechanical loading, disc have limited capacity for self-repair and are vulnerable to damage (Huang et al., 2013).

Abnormal mechanical stresses, nutritional deficiencies, and the aging process are recognized as inductive factors for regulated cell death (RCD) in intervertebral disc cells (Vergroesen et al., 2015; Yang et al., 2022; Yang et al., 2019; Kang et al., 2020a). This cellular demise leads to the disruption of the normal architectural and physiological functions of the disc, ultimately progressing to intervertebral disc degeneration (IVDD) (Kepler et al., 2013; Walter et al., 2011). IVDD further serves as a primary contributor to a multitude of spinal disorders that pose a significant global health burden (Liebsch and Wilke, 2022; Binch et al., 2021). The escalating incidence of IVDD is poised to exacerbate the global prevalence of pain, disability, and the associated economic strain on healthcare systems (Collaborators, 2021; GBD, 2019 Diseases and Injuries Collaborators, 2020; GBD 2017 Disease and Injury Incidence and Prevalence Collaborators, 2018).

Mitochondria serve as the “powerhouse” of cells, generating energy in the form of ATP and participating in various vital cellular processes (Dc, 2013). However, mitochondria are prone to damage, leading to mitochondrial dysfunction and imbalance of cell homeostasis, which are closely associated with the occurrence of various diseases (Doblado et al., 2021). Thus, maintaining mitochondrial homeostasis is of vital importance. Mitophagy selectively eliminates damaged mitochondria and maintains their quality stability. On one hand, mitophagy is capable of selectively identifying and removing damaged mitochondria to prevent them from causing further harm to cells. On the other hand, through mitophagy, cells can adjust the quantity and quality of mitochondria to adapt to different metabolic requirements and microenvironmental changes (Pickles et al., 2018). This contributes to ensuring that cells function optimally in different physiological and pathological conditions.

Mitochondria play a crucial regulatory role in skeletal muscle physiology, demonstrating stimulus-responsive alterations in quantity, configuration, and performance under external stress conditions (Wu et al., 2024). The metabolic functions of mitochondria in hypoxic intervertebral disc environments have been largely overlooked (Madhu et al., 2020). It is only in recent years that steady advancements have been made in understanding the association between mitophagy and IVDD, revealing a close correlation between maintaining a healthy mitochondrial pool and preventing IVDD (Lin et al., 2023). Emerging research findings have facilitated the development of novel diagnostic protocols and more targeted interventions (Vlaeyen et al., 2018). Due to the distinct structures, physiological functions, and microenvironments of the NP, AF, and CEP, the pathological processes and repair mechanisms following injuries to these regions exhibit region-specific characteristics (Kepler et al., 2013; Xu et al., 2024). This heterogeneity profoundly impacts therapeutic targeting, as each subregion faces unique mitochondrial challenges, necessitating tailored strategies. In this review, we will summarize the mechanisms of mitophagy in distinct regions of the intervertebral disc, and discuss therapeutic strategies employing mitophagy modulators to delay IVDD. The goal is to provide significant insights that are broadly pertinent to enhancing human health and quality of life for patients suffering from related conditions.

## 2 Methods

### 2.1 Information sources and search strategies

A literature search was conducted in the PubMed from inception to March 2025. The keywords “nucleus pulposus” (6,796), “annulus fibrosus” (2,366), “cartilage endplate” (1,671), “intervertebral disc” (43,493), and “intervertebral disc degeneration” (13,699) were independently searched and then combined with the terms “mitophagy” (Liebsch and Wilke, 2022; Li et al., 2019), “mitochondria” (268,773), “mitochondrial homeostasis” (27,901), and “mitochondrial dysfunction” (103,290). Specifically, the combined searches retrieved 225 results. The reference lists of relevant studies were additionally screened to identify potentially eligible articles. The potentially eligible studies were then screened by three independent authors (C.F., Z.H., and M.Z.). The screenings were cross-checked, and any discrepancies were resolved through discussion with a senior reviewer (X.F.). After this process, 23 articles were ultimately included.

### 2.2 Eligibility criteria

Studies were selected according to the following criteria.

Inclusion criteria: 1) Original research articles investigating NP, AF, or CEP in vitro or *in vivo* models; 2) Studies that explicitly assessed mitophagy phenotypes.

Exclusion criteria: 1) Duplicate publications or studies with overlapping datasets; 2) Articles lacking direct experimental evidence on NP, AF, or CEP.

## 3 Results

### 3.1 Development of mitophagy in IVDD

**FIGURE 1:**
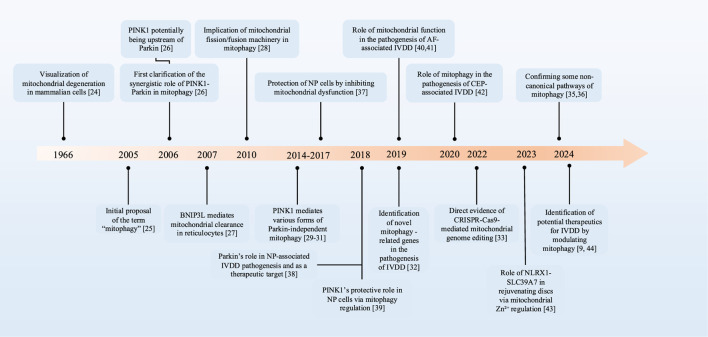
The development timeline of mitophagy in IVDD. PTEN, phosphatase and tensin homologue; PINK1, PTEN-induced putative kinase 1; BNIP3L, BCL2-interacting protein 3-like; IVDD, intervertebral disc degeneration; NP, nucleus pulposus; AF, annulus fibrosus; CEP, cartilage endplate.

During the past few decades, the research on mitophagy has demonstrated consistent progress, and its exploration in IVDD has exhibited a notable development trend over the recent years (Figure 1).

In the 1960s, based on the findings of electron microscope studies, sufficient evidence existed to demonstrate the degradation of mitochondria and other intracellular structures in lysosomes within mammalian cells (Duve and Wattiaux, 1966). The term “mitophagy” was initially proposed to delineate the process of selective autophagy of mitochondria in 2005, highlighting its role as a targeted defense mechanism (Lemasters, 2005). As the research advanced, the synergistic regulation of mitophagy by PTEN induced putative kinase 1 (PINK1) and Parkin was first elucidated in the experiment, and studies suggest that PINK1 may be upstream of Parkin in the regulatory pathway (Clark et al., 2006). Additionally, the BCL2-interacting protein 3-like (BNIP3L) receptor, also known as Nip3-like protein X (NIX) receptor, has also been proven to play a crucial role in the selective elimination of mitochondria (Schweers et al., 2007). By the 2010s, research into mitophagy mechanisms had advanced significantly. Researchers have not only revealed a strong link between mitochondrial fission, fusion mechanisms, and mitophagy (Ding et al., 2010), but also, in 2014, identified that PINK1 possesses additional phosphorylation sites, which are capable of compensating for the function of Parkin in mitophagy (Dave et al., 2014; Lai et al., 2015; Villa et al., 2017). Over the following years, multiple PINK1-mediated mitophagy processes that are independent of Parkin have been further validated and studied. With advancements in gene editing and high-throughput sequencing technologies, novel mitophagy-related genes have been identified (Xie et al., 2019). Particularly, CRISPR-Cas9-mediated mitochondrial genome editing has enabled successful mtDNA modification while systematically identifying key regulators of mitophagy (Bi et al., 2022). Recent research has demonstrated the existence of machinery capable of delivering mitochondrial constituents and membranes to lysosomes in the absence of autophagy. For instance, inner mitochondrial membrane (IMM)-mediated mitophagy and mitochondrial extracellular vesicles (mitoEVs) are recognized as mechanisms for delivering mitochondrial components to lysosomes, thereby compensating for the deficiencies in canonical mitophagy (Konig et al., 2021; Saunders et al., 2024; Iorio et al., 2024).

In the research on mitophagy and IVDD, a study in 2017 pointed out that mitochondrial dysfunction is associated with the apoptosis of NP cells, suggesting that improving mitochondrial dysfunction could be a new way for effectively protecting NP cells (Xu et al., 2017). Subsequently, scientists discovered that Parkin is involved in the pathogenesis of IVDD and may serve as a potential therapeutic target for IVDD (Zhang et al., 2018). The crucial role of PINK1 in eliminating damaged mitochondria and alleviating the senescence of NP cells through the mitophagy pathway was also revealed (Y et al., 2018). In 2019, studies focused on the role of mitochondrial function in the pathogenesis of AF-related IVDD (Wu et al., 2021; Xu W-N. et al., 2019). In 2020, the importance of Parkin-mediated mitophagy in the survival of CEP cells under pathological conditions was unveiled (Kang et al., 2020b). In 2023, the role of the NLRX1-SLC39A7 complex in orchestrating mitochondrial dynamics and mitophagy to rejuvenate intervertebral disc through modulation of mitochondrial Zn^2+^ trafficking was unveiled (Song et al., 2024). These findings have provided new insights into understanding the pathological mechanisms and potential therapeutic approaches for IVDD. Concurrently, certain medications have exhibited potential in addressing IVDD by regulating mitophagy levels, bringing new therapeutic options and directions insights for future research and treatment (Kang et al., 2020a; Lin et al., 2020).

### 3.2 Molecular mechanisms of mitophagy

This primarily procedure of mitophagy consists of several sequential events: Firstly, damaged mitochondria depolarize and lose membrane potential. Secondly, mitochondria are wrapped by autophagosomes to form mitochondrial autophagosomes. Thirdly, these mitochondrial autophagosomes fuse with lysosomes. Lastly, the contents of the mitochondria are degraded by lysosomes (Xu et al., 2020).

**FIGURE 2:**
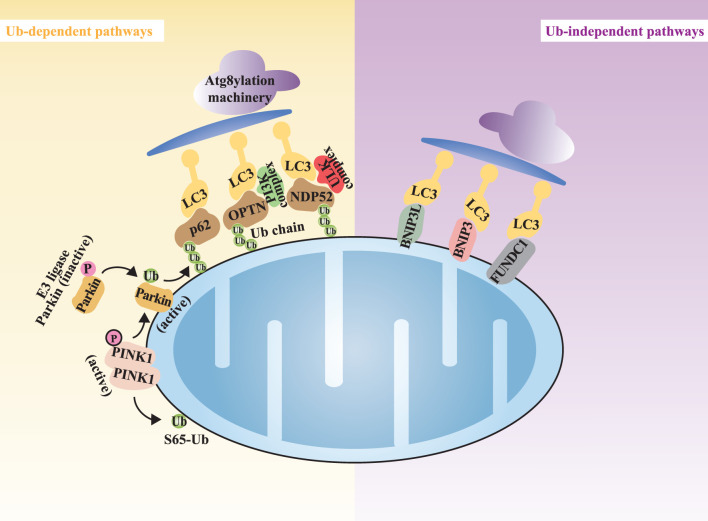
The overview of the mitophagy mechanisms. Note: Mitophagy can be categorized into Ub-dependent pathways (highlighted in yellow) and Ub-independent pathways (highlighted in purple). Atg8yl-Mach, Atg8ylation machinery; OPTN, optineurin; NDP52, nuclear dot protein 52; LC3, microtubule-associated protein1 light chain 3.

In diverse cellular environments, various stimuli can induce mitophagy through multiple signaling cascades (Palikaras et al., 2017). Mitophagy operates through distinct yet interrelated mechanisms. These mechanisms can generally be categorized into ubiquitin (Ub)-dependent and Ub-independent pathways (Khaminets et al., 2016). The Ub-dependent pathways are further divided into the Parkin-dependent and Parkin-independent pathways (Birgisdottir et al., 2013; Chen G. et al., 2020) (Figure 2).

#### 3.2.1 Ub-dependent pathways

The Ub-dependent pathways rely on extensive ubiquitination of damaged mitochondrial surface proteins to promote mitophagy. In the field of Ub-dependent mitophagy, the Parkin-dependent pathway, mediated by the kinase PINK1 and the E3 ubiquitin ligase Parkin, is the most extensively studied mechanism (Clark et al., 2006; Ashrafi and Schwarz, 2013). PINK1, a highly conserved mitochondrial protein encoded by the PARK6 gene, is involved in regulating various cellular physiological processes, particularly crucial for the mitochondrial function (Wang N. et al., 2020). In healthy mitochondria, PINK1 is continuously imported into the IMM and degraded, maintaining low expression levels (Narendra et al., 2010; Jin et al., 2010; Yamano and Youle, 2013). However, when mitochondrial membrane potential (MMP) is compromised, leading to mitochondrial dysfunction, the import channel for PINK1 into the IMM is blocked, causing its accumulation at the translocase complex on the outer mitochondrial membrane (OMM) of the damaged mitochondria [45, 46]. At this location, PINK1 undergoes dimerization, triggering autophosphorylation and activation (Gan et al., 2022; Rasool et al., 2022). The activated PINK1 subsequently phosphorylates serine 65 of Ub and the Ub-like domain of Parkin, resulting in the further categorization of Ub-dependent mitophagy into Parkin-dependent and Parkin-independent pathways (Uoselis et al., 2023). In the Parkin-dependent pathway, Parkin, encoded by the PARK2 gene, is responsible for conjugating Ub-molecules to substrates (Riley et al., 2013). Autophagy receptor proteins, such as optineurin (OPTN) and nuclear dot protein 52 (NDP52), play a pivotal role in the PINK1/Parkin pathway (Lazarou et al., 2015; Heo et al., 2015; Wong and Holzbaur, 2014). These autophagy receptor proteins bind to ubiquitinated substrates and associate with ATG8 family members, facilitating the capture of damaged mitochondria by autophagosomes through the Atg8ylation machinery (Atg8yl-Mach). The Atg8yl-Mach is composed of the ATG12-ATG5-ATG16L1 complex, which anchors ATG8 to the autophagosome membrane by promoting its conjugation to phosphatidylethanolamine, thereby promoting the formation and expansion of autophagosomes on the surface of damaged mitochondria. Members of the ATG8 family are divided into the microtubule-associated protein one light chain 3 (LC3) and GABA receptor-associated protein (GABARAP) subgroups. During the downstream process of mitophagy initiation, ATG8 family members play a crucial role in the fusion of autophagosomes with lysosomes for the degradation of mitochondrial substrates, a process primarily driven by the GABARAP subgroup (Nguyen et al., 2016; Vaites et al., 2017).

In addition to the classical PINK1/Parkin pathway, there exist other Ub-dependent pathways that are independent of Parkin. In Parkin-independent mitophagy, activated PINK1 can directly recruit autophagy receptor proteins to mitochondria by phosphorylation of Ub at the serine 65 site. Next, the Ub chains generated on OMM substrates serve as recruitment platforms for Ub-binding autophagy receptor proteins, including sequestosome one/p62 (SQSTM1/p62), neighbor of BRCA1 (NBR1), OPTN, NDP52, and Tax1-binding protein 1 (TAX1BP1). These receptor proteins function by initiating the formation of autophagosomes (Lazarou et al., 2015; Richter et al., 2016). The aforementioned OMM autophagy-related proteins contain both LC3-interacting region (LIR) and Ub-binding domain (UBD), thereby mediating the binding of LC3 to Ub chains on targeted mitochondria. As a result, these proteins anchor ubiquitinated mitochondria to autophagosomes (Fan et al., 2021).

#### 3.2.2 Ub-independent pathways

Ub chains are not the only factor recruiting autophagy receptor proteins. The autophagy receptor proteins on the OMM inherently possess LIR. These autophagy receptors can directly bind to LC3 without ubiquitination, thus initiating mitophagy. In mammals, such receptors primarily include NIX receptor, BCL2-interacting protein 3 (BNIP3) receptor, and FUN14 domain-containing protein 1 (FUNDC1) receptor, among others (Lu et al., 2023) (Figure 2).

NIX and BNIP3 share 56% homology and both contain the BCL2 homology 3 (BH3) domain, which allows them to directly bind to LC3 through their BH3 domains and induce mitophagy (Novak et al., 2010). FUNDC1 can interact with LC3 to induce Parkin-independent mitophagy under hypoxic conditions (Liu et al., 2012).

#### 3.2.3 Non-canonical pathways

Non-canonical autophagy, independent of autophagosome formation, represents an endosomal-dependent mitophagy pathway activated under specific stress conditions. Specifically, upon mitochondrial DNA damage, mitochondrial nucleoids are eliminated via the endosome-mitophagy pathway. MitoEVs that bud from mitochondrial networks have been implicated as a means of delivering mitochondrial components to lysosomes (Konig et al., 2021; Iorio et al., 2024; Soubannier et al., 2012), thereby compensating for deficiencies in canonical mitophagy (Towers et al., 2021). In addition to mitoEVs-mediated mitophagy, mitochondrial herniation leads to the exposure and ubiquitination of the IMM, initiating the induction of an apoptotic mitophagy sequestration pathway. IMM-mitophagy has the potential to capture herniating mitochondria, theoretically preventing mtDNA release into the cytosol at an earlier stage of the process before any transcriptional response can be triggered. Using proximity proteomics, researchers have also identified the protein required for the clearance of mutated mitochondrial nucleoids from the mitochondrial matrix. Among these, ATAD3 and SAMM50 regulate both the architecture of mitochondrial cristae and nucleoid interactions. SAMM50 cooperates with the retromer complex protein VPS35 to sequester mitochondrial DNA within endosomes, thereby preventing excessive immune response (Sen et al., 2023).

## 4 Role of mitophagy in IVDD and therapeutic approaches

### 4.1 Role of mitophagy in IVDD

#### 4.1.1 Mitophagy in NP region of IVDD

**FIGURE 3:**
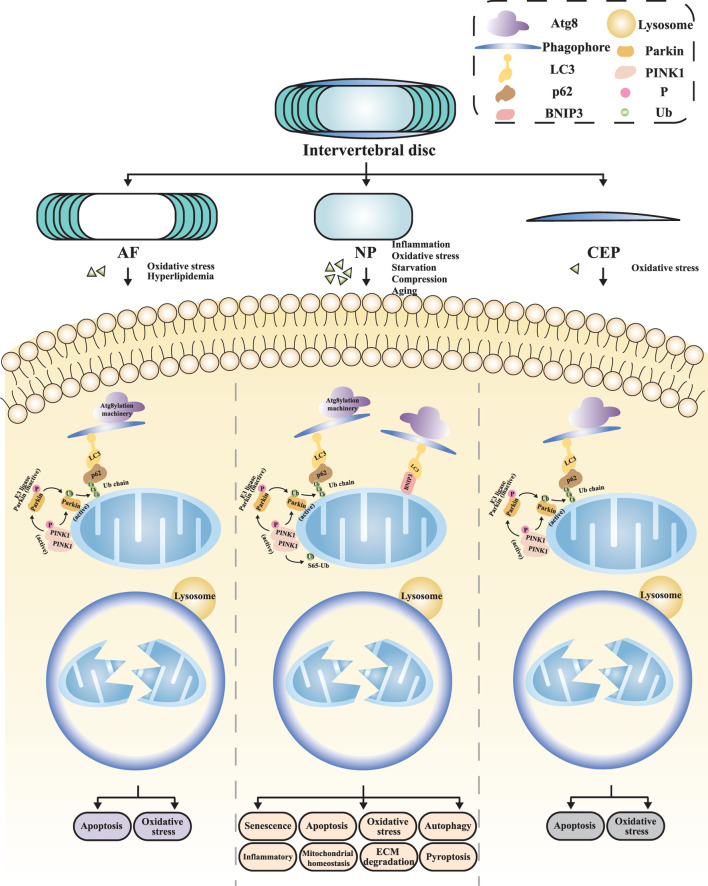
The mitophagy in distinct regions of IVDD NP, nucleus pulposus; AF, annulus fibrosus; CEP, cartilage endplate; ECM, extracellular matrix.

The complex multi-tissue structure of the intervertebral disc allows it to absorb and distribute mechanical stresses during physical activities (Konig et al., 2021). Specifically, the NP is primarily composed of NP cells and extracellular matrix (ECM), with the ECM of the NP consisting of type II collagen and proteoglycans (Risbud et al., 2015). NP cells maintain the biomechanical homeostasis of the NP by synthesizing and secreting ECM (Vamvakas et al., 2017; Lin J. et al., 2019; Silagi et al., 2018). Nutrients and metabolites enter and exit the disc through diffusion within the dense ECM (Wise et al., 2020). Evidence suggests that IVDD initially occurs in the NP region of the disc (Guerrero et al., 2021), and research on the mechanisms and therapeutic strategies of IVDD has predominantly focused on NP cells (Xin et al., 2022; Wu et al., 2022; Zhang et al., 2021; Sun et al., 2022). Currently, the etiology of RCD induced by mitochondrial dysfunction in NP cells involves multiple factors, including inflammation, oxidative stress, nutrient deficiency, compression, and hyperlipidemia (Figure 3).

Firstly, inflammation is considered a significant pathogenic factor. Pro-inflammatory cytokines, such as interleukin-1β (IL-1β), tumor necrosis factor-α (TNF-α), and interleukin-6 (IL-6), collectively accelerate the progression of IVDD by promoting ECM degradation, chemokine production, immune cell recruitment, and phenotypic changes in disc cells (Xu et al., 2017; Zhang et al., 2018; Risbud and Shapiro, 2014; Peng et al., 2022). The relationship between inflammation induction and mitophagy has garnered attention from Zhang and his team (Zhang et al., 2018). They made pioneering observations that Parkin expression is not only elevated in degenerated human NP but also increased in rat NP stimulated with TNF-α. Meanwhile, TNF-α stimulates NP cells to produce more reactive oxygen species (ROS), subsequently activating autophagy and apoptosis processes. During this process, despite increased expression levels of LC3 and Beclin-1, p62 levels also rise in NP cells, indicating impaired autophagy flux. Dysfunction in Parkin-dependent mitophagy has been confirmed as a pivotal cause. Further research has found that the NF-κB signaling pathway is a potential mechanism through which pro-inflammatory cytokines exert their effects. Studies by Zhao et al. (2020) and Yu et al. (2021) demonstrated that the inflammation induced by TNF-α can affect mitochondrial function in NP cells through the NF-κB pathway, which in turn triggers a series of secondary phenotypic changes, including exacerbated inflammation, oxidative stress, and pyroptosis. Among these, infiltration and activation of immune cells further amplify the inflammatory cascade, leading to aggravated inflammation (Risbud and Shapiro, 2014). Cytokines induce oxidative stress by increasing ROS accumulation (Yang et al., 2023). Furthermore, pyroptosis is dependent on inflammasome activation and is accompanied by the massive release of inflammatory cytokines. Evidence suggests that activated NLRP3 inflammasomes aggregate around mitochondria, and their potential detrimental effects in IVDD have attracted widespread attention (Zhou et al., 2011; Xia et al., 2019). NLRP3 inflammasome induces pyroptosis and release inflammatory cytokines in NP cells, a process that promotes the secretion of metalloproteinases and leads to NP degradation (Song et al., 2017; A et al., 2020), thereby accelerating the pathological progression of IVDD (Tang et al., 2021). Research by Peng et al. (Peng et al., 2022) found that in a lipopolysaccharide (LPS) induced inflammation model, activation of the NLRP3 inflammasome promotes ROS production and inhibits mitophagy flux. This leads to pyroptosis and apoptosis of NP cells, resulting in accelerated degeneration of the intervertebral disc NP.

Secondly, mitochondria are both the primary source of cellular ROS and highly susceptible to oxidative stress damage, leading to dysfunction (Hm et al., 2015). Impaired mitophagy will result in mitochondrial dysfunction and ROS accumulation (Zhang et al., 2018; Lin Q. et al., 2019). ROS accumulation, in turn, leads to exaggerated inflammation, disordered metabolism, and enhanced apoptosis in cells (Zhou et al., 2011). Among the molecules triggered by mitochondrial ROS, the NLRP3 inflammasome has been extensively studied for its detrimental role in IVDD (Zhou et al., 2011; Xia et al., 2019). Activation of the NLRP3 inflammasome can elevate the production of IL-1β, which facilitates the secretion of metalloproteinases and subsequently causes degradation of NP tissue (Song et al., 2017; A et al., 2020). Furthermore, NLRP3 is linked to the mitochondrial apoptosis pathway, programmed cell death, and apoptosis through several mechanisms in NP cells (Wn et al., 2019). In Wang et al.‘s (Y et al., 2018) study, treatment of human NP cells with H_2_O_2_ led to impaired mitophagy, manifesting as ROS accumulation, decreased ECM synthesis, and accelerated senescence. These alterations collectively contributed to the degeneration of NP cells. Additionally, tert-butyl hydroperoxide (TBHP) is also widely used to simulate oxidative stress environments. Various scholars (Xie et al., 2019; Lin et al., 2020; Wang et al., 2018; Wn et al., 2019; Chen et al., 2019; Chen Y. et al., 2020) have explored the role of mitophagy in TBHP-induced oxidative stress using different experimental models, finding that TBHP treatment results in impaired mitophagy in NP cells of the intervertebral disc. This includes both the Ub-dependent classical pathway, characterized by decreased expression levels of PINK1 and Parkin proteins, reduced LC3 II/I ratio, and decreased MMP and ATP levels, as well as BNIP3-mediated Ub-independent mitophagy (Wang et al., 2018). BNIP3, initially identified as a pro-apoptotic protein, features an atypical BH3 domain localized to the OMM. Induction of BNIP3 triggers the translocation and activation of BCL2-antagonist/killer 1 (BAK1) and Bcl-2 Associated X Protein (BAX) to mitochondria, leading to increased mitochondrial membrane permeability, subsequent release of cytochrome C from mitochondria to the cytosol, and ultimately initiating the caspase cascade of apoptosis. Emerging research has found that BNIP3 also serves as a key receptor for mitophagy, playing a role in promoting cell survival (Madhu et al., 2020). The bidirectional functions of BNIP3 in apoptosis and mitophagy suggest that it may be a critical regulator of cell fate (Madhu et al., 2023). The aforementioned changes in mitophagy will further affect processes such as apoptosis, senescence, ROS generation, and ECM degradation, ultimately influencing the degeneration of the NP of the intervertebral disc.

In addition to inflammation and oxidative stress, nutrient deficiency and abnormal mechanical loads are also key risk factors for IVDD. Wang et al. (2020b) found that nutrient deficiency and aging can downregulate FOXO3, leading to mitochondrial dysfunction and inhibited mitophagy, resulting in increased NP cell apoptosis and ECM degradation. Abnormal compressive forces, tensile forces, and increased matrix stiffness all exert detrimental effects on disc cells (Wang D. et al., 2022; Xiao et al., 2022; Wang et al., 2021). Further research has shown that excessive mechanical stress applied to NP cells can also lead to oxidative stress and mitochondrial dysfunction (Kang et al., 2020a; Hu et al., 2022). Kang et al. (Kang et al., 2020a) found that mechanical compression can cause mitochondrial dysfunction in NP cells of the intervertebral disc, with increased ROS production, exacerbated mitochondrial dysfunction, and increased apoptosis, thereby promoting NP cell degeneration. Mitochondrial dysfunction can further increase ROS production, leading to a vicious cycle between mitochondrial dysfunction and ROS accumulation, causing sustained oxidative damage (Chen et al., 2018).

Combining the above evidence, mitochondrial damage leads to pathological phenotypes in NP cells, including apoptosis, oxidative stress, senescence, ECM degradation and synthesis, excessive autophagy, exacerbated inflammation, and pyroptosis (Table 1).

**TABLE 1:** The role of mitophagy in IVDD.

Authors, year	Target cell/tissue	Stressors	Phenotypes	Mitophagy pathway	Disease effect
NP					
Zhang et al. (2018)	Rat NP cells, Rat *in vivo*	TNF-α	ROS generation↑ Autophagy# Apoptosis↑	Parkin-mediated mitophagy	IVDD
Y et al. (2018)	Human NP cells	H_2_O_2_	ROS generation↑ ECM synthesis↓ Autophagy# Senescence↑	Parkin-mediated mitophagy	IVDD
Wang et al. (2018)	Rat NP cells Rat *in vivo*	TBHP	ROS generation↑ Senescence↑ Apoptosis↑	Ub-independent pathways (BNIP3)	IVDD
Xie et al. (2019)	Rat NP cells Rat *in vivo*	TBHP	ECM degradation↑ Senescence↑ Apoptosis↑	Parkin-mediated mitophagy	IVDD
Xu et al., (2019b)	Rat NP cells Rat *in vivo*	TBHP	ROS generation↑ Excessive autophagy↑ Apoptosis↑	Parkin-mediated mitophagy	IVDD
Chen et al. (2019)	Rat NP cells Rat *in vivo*	TBHP	ROS generation↑ ECM degradation↑ Apoptosis↑	Parkin-mediated mitophagy	IVDD
Chen et al. (2020b)	Rat NP cells Rat *in vivo*	TBHP	ROS generation↑ Apoptosis↑	Parkin-mediated mitophagy	IVDD
Wang et al. (2020b)	Rat NP cells	Starvation Aging	ECM degradation↑ Apoptosis ↑	Parkin-mediated mitophagy	IVDD
Lin et al. (2020)	Rat NP cells Rat *in vivo*	TBHP	Autophagy↓ Apoptosis↑	p62↑	IVDD
Kang et al. (2020a)	Human NP cells Rat NP tissue	Compression	ROS generation↑ Apoptosis↑	Parkin-mediated mitophagy	IVDD
Peng et al. (2022)	Rat NP cells	LPS	ROS generation↑ Pyroptosis↑ Apoptosis↑	p62↑	IVDD
Madhu et al. (2023)	Rat NP cells Mouse *in vivo*	LV-shBNIP3	ECM synthesis↓ Metabolic homeostasis↓	Ub-independent pathways (BNIP3)	IVDD
Song et al. (2024)	Human NP cells Mouse NP cells Rat *in vivo*	TBHP	Excessive autophagy↑ Senescence↑	Parkin-mediated mitophagy	IVDD
Gu et al. (2024)	Human NP cells Rat *in vivo*	IL-1β	ECM synthesis↓ ROS generation↑ ECM synthesis↓ Senescence↑ Apoptosis↑	Parkin-mediated mitophagy	IVDD
AF					
Xu et al. (2019a)	Rat AF cells, Rat *in vivo*	TBHP	Oxidative stress↑ Apoptosis↑	Parkin-mediated mitophagy	IVDD
CEP					
Kang et al. (2020b)	Human CEP cells Rat CEP cells	H_2_O_2_	Oxidative stress↑ Apoptosis↑	Parkin-mediated mitophagy	IVDD

Note: “↑” for upregulation (increased expression); “↓” for downregulation (decreased expression); “#” for activated autophagy with impaired flux.

NP, nucleus pulposus; AF, annulus fibrosus; CEP, cartilage endplate; ECM, extracellular matrix; TBHP, tert-butyl hydroperoxide; ROS, reactive oxygen species; IVDD, intervertebral disc degeneration; BNIP3, BCL2-interacting protein 3; LPS, lipopolysaccharide; LV: lentivirus.

#### 4.1.2 Mitophagy in AF region of IVDD

As the outer structure of the intervertebral disc, the primary physiological function of the AF lies in its ability to effectively encapsulate the NP, preventing its herniation through its unique hydraulic sealing properties, and evenly distributing the various pressures acting on the disc (Moore, 2006). The AF comprises two distinct components: the inner AF, which is adjacent to the NP and consists of chondrocytes with an ECM primarily composed of type II collagen; and the outer AF, which is mainly composed of fibroblast-like cells with an ECM primarily composed of type I collagen. This lamellar structure provides the intervertebral disc with high flexibility and adaptability across multiple planes of motion, ensuring the stability and normal function of the disc structure (Smith et al., 2011; Roughley, 2004). The outer AF receives nutritional support from capillaries within the surrounding soft tissues, while the rest of the AF exchanges nutrients and metabolic waste through a capillary network at the CEP via diffusion (Wise et al., 2020) (Figure 3). Due to the unique structure and physiological function of the AF, the pathological repair process following AF injury exhibits distinct characteristics (Bailey et al., 2013). Clinically, both acute trauma and chronic degeneration of the AF can exacerbate IVDD. Studies have demonstrated that AF injury contributes to disc instability and disrupts the intradiscal microenvironment. Meanwhile, AF injuries often persist due to insufficient endogenous repair capacity [105]. Histologically, AF scar healing is predominantly characterized by disorganized type III collagen deposition, with sparse type I collagen bundles observed in the outer layer. The key subsequent effects include reduced resistance to small molecule permeation, decreased tensile strength, diminished disc height, NP fibrosis, and CEP subchondral ossification (Kuivaniemi and Tromp, 2019). These findings underscore the necessity of elucidating AF repair mechanisms and their systemic impact on disc homeostasis.

Oxidative stress and abnormal lipid metabolism are currently recognized as significant factors inducing IVDD through mitophagy-mediated AF damage. Xu et al. (Konig et al., 2021) demonstrated that TBHP-induced oxidative stress can lead to mitochondrial dysfunction accompanied by downregulation of mitophagy levels. When mitophagy function is impaired, oxidative stress and apoptosis levels significantly increase, accelerating the occurrence and progression of IVDD. Further research revealed that SIRT2, upstream of this pathway, is a key target that influences the expression levels of peroxisome proliferator-activated receptor γ coactivator 1α (PGC-1α), Parkin, and LC3 II, thereby regulating mitophagy. Wu et al. (Wu et al., 2021) found that oxidized low-density lipoprotein (oxLDL) can promote mitochondrial fission, further exacerbating mitochondrial dysfunction and increasing AF cell apoptosis, thereby accelerating the pathological process of IVDD.

In brief, oxidative stress and abnormal lipid metabolism are crucial factors inducing IVDD through mitophagy-mediated AF damage. The pathological phenotypes of AF cells resulting from mitochondrial damage include apoptosis and oxidative stress (Table 1).

#### 4.1.3 Mitophagy in CEP region of IVDD

The CEP consists of a biological tissue layer rich in type II collagen and chondrocytes, situated between the intervertebral disc and adjacent vertebral body. It is similar to other articular tissues in the body, and it exhibits the highest cellular density among all structures of the intervertebral disc (Kirnaz et al., 2022). The CEP serves as the primary pathway for nutrient delivery from vertebral body capillaries to the disc, as well as for the excretion of waste products from the disc. Degeneration of the CEP can hinder the nutrition and waste exchange of the intervertebral disc, leading to the disruption of its homeostasis and the initiation of IVDD (Wong et al., 2019). Kang et al. (Kang et al., 2020b) investigated the regulatory role of oxidative stress on mitophagy mechanisms in CEP cells. Their study induced an oxidative stress state with H_2_O_2_, resulting in mitochondrial dysfunction manifested as decreased MMP, reduced ATP synthesis, increased ROS levels, and opening of the mitochondrial permeability transition pore (mPTP). Concurrently, mitophagy was inhibited, further exacerbating cellular apoptosis and ultimately accelerating the progression of IVDD (Figure 3).

In summary, impaired mitophagy function leads to a series of pathological phenotypes in CEP cells, including enhanced apoptosis and oxidative stress. Additionally, this mechanism has been extensively studied in other articular cartilage tissues structurally similar to CEP cells and has been shown to be closely related to the pathological processes of these tissues (Sun et al., 2021) (Table 1).

Mitophagy, as an important mechanism for cellular self-renewal and homeostasis maintenance, exhibits significant correlations with various forms of RCD (Sun et al., 2018; Sun et al., 2019). It is important to note that mitophagy is a double-edged sword. Moderate mitophagy can protect cells from various external stimuli, whereas excessive mitophagy can also accelerate cellular apoptosis and the progression of IVDD (Kang et al., 2020a; Xu W-N. et al., 2019).

### 4.2 Therapeutic strategies for targeting mitophagy in IVDD

Intervertebral disc cells reside in a physically avascular and hypoxic microenvironment, primarily relying on anaerobic glycolysis for energy production (Urban et al., 2004). Based on this observation, it was once widely accepted in the academic community that, compared to cells dependent on aerobic metabolism, intervertebral disc cells contain fewer functional mitochondria (Madhu et al., 2020; Gan et al., 2003). However, subsequent studies have revealed the presence of a functional mitochondrial network within NP cells, capable of adjusting mitochondrial quantity through active mitochondrial flux to match metabolic demands. Furthermore, intervertebral disc metabolism is relatively active, and its internal cells, due to oxygen scarcity, have developed compensatory mechanisms to counteract relative hypoxia, including upregulation of HIF-1α and others (Risbud et al., 2010; Theodore, 2020). Additionally, mitochondrial dysfunction and abnormal mitochondrial morphology can be observed in degenerated intervertebral disc cells (Hu et al., 2022; Song et al., 2018). Consequently, therapeutic strategies aimed to ameliorate IVDD through modulation of mitophagy have emerged as a focal point of current research, increasingly gaining attention and recognition from scholars in the field.

#### 4.2.1 Pharmacological interventions

Numerous natural products exhibit therapeutic potential for improving IVDD by modulating mitophagy levels. Hydrogen sulfide (H2S), along with nitric oxide and carbon monoxide, is regarded as one of three endogenously produced gaseous signaling molecules. These molecules possess diverse biological functions, including anti-inflammatory and anti-apoptotic effects, and exert impacts on multiple key mechanisms and pathways both *in vivo* and *in vitro* (Hu et al., 2007; Hu et al., 2009). Recent studies have demonstrated that H_2_S effectively improves mitochondrial function by closing the mPTP, enhancing MMP, and ATP levels, thereby reducing cellular apoptosis and showing therapeutic potential for IVDD (Xu et al., 2017). Salidroside, a phenylpropanoid glycoside extracted from Rhodiola, and Polydatin, a resveratrol glycoside extracted from the rhizomes of Polygonum cuspidatum, can both activate mitophagy through a Parkin-dependent pathway, upregulate Parkin protein expression, promote the reduction of ROS accumulation, and effectively inhibit cellular apoptosis, thereby ameliorating mitochondrial damage and apoptosis in NP and CEP cells, respectively (Zhang et al., 2018; Kang et al., 2020b). Notably, Polydatin can also simultaneously activate the Nrf2 pathway, upregulating Nrf2 protein expression and its nuclear translocation, further improving mitochondrial dysfunction (Kang et al., 2020b). Urolithin A, a metabolite of ellagitannins and ellagic acid abundant in pomegranates, strawberries, and other nuts (Cerdá et al., 2005), can specifically induce mitophagy both *in vivo* and *in vitro* (Ryu et al., 2016; Fang et al., 2019). Mechanistic studies have shown that Urolithin A inhibits NP cell apoptosis by activating mitophagy through the AMPK pathway, thereby slowing down the progression of IVDD (Lin et al., 2020). Honokiol, a natural flavonoid compound derived from the roots and bark of Magnolia officinalis, also exerts therapeutic effects by activating the AMPK pathway. Honokiol demonstrates multiple pharmacological effects such as antioxidant, anti-lipid peroxidation, anti-inflammatory, and neuroprotective activities, showing therapeutic potential in cartilage protection and IVDD (Chen et al., 2014; Chen et al., 2015). To explore the mechanism of Honokiol-induced SIRT3 expression enhancement, Wang et al. (Wang et al., 2018) examined the activation of two major energy sensor molecules, namely, AMPK and PGC-1α. By activating the AMPK pathway, upregulating PGC-1α and SIRT3, regulating mitochondrial dynamics, and enhancing mitophagy flux through the autophagy receptor BNIP3, Honokiol protects NP cells from oxidative stress damage, reversing the processes of aging and apoptosis (Wang et al., 2018). Furthermore, natural isothiocyanate compounds such as Sulforaphane, found in cruciferous vegetables, especially broccoli, have been shown to improve mitochondrial dysfunction, reduce mitochondrial morphological abnormalities, and enhance mitochondrial dynamics. The specific mechanisms involve upregulating PGC-1α expression and AMPK phosphorylation, reducing ROS accumulation, delaying aging, inhibiting apoptosis, and reducing ECM degradation (Xu X. et al., 2019). Mangiferin exhibits potent free radical scavenging activity, with mango trees serving as its primary and readily accessible source (Zhao et al., 2017). It possesses multiple pharmacological potentials, including antioxidant, anti-inflammatory, anti-diabetic, anti-hyperlipidemic, and anti-atherosclerotic properties, with mechanisms involving the counteraction of oxidative stress and mitochondrial dysfunction (Alberdi et al., 2018; Li et al., 2019). Opa1, Drp1, and TFAM are biomarkers of mitochondrial dynamics. Mangiferin not only downregulates Drp1 expression but also upregulates Opa1 and TFAM levels, thereby reducing inflammation, ECM degradation, oxidative stress, and apoptosis (Yu et al., 2021). Selenium can also reduce oxidative stress and cellular apoptosis by regulating mitochondrial dynamics and the expression of autophagy-related proteins (Wang P. et al., 2022) (Table 2).

**TABLE 2:** Therapeutic strategies for IVDD through mitochondrial homeostasis regulation.

Category	Treatment	Region	Regulator	Mitochondrial autophagy/homeostasis	Phenotype	References
Natural ingredient	H_2_S	NP	N/A	Function:mPTP↓, MMP↑, ATP↑	Apoptosis↓	Xu et al. (2017)
Natural ingredient	Salidroside	NP	N/A	Function:ΔΨm↑, MMP↑ Mitophagy:Parkin↑	Autophagy↑ Oxidative stress↓ Apoptosis↓	Zhang et al. (2018)
Natural ingredient	Honokiol	NP	AMPK/PGC-1α/SIRT3↑	Mitochondrial dynamics:Drp1↑, Fis1↑, Mfn2↑ Mitophagy:BNIP3↑, LC3 II/I↑	Oxidative stress↓ Senescence↓ Apoptosis↓	Wang et al. (2018)
Natural ingredient	Sulforaphane	NP	AMPK/PGC-1α↑ Progerin↓	Function:ΔΨm↑, ATP↑ Mitochondrial dynamics: Drp1↓, Mfn1/2↑	Oxidative stress↓ Senescence↓ Apoptosis↓ ECM degradation↓	Xu et al. (2019b)
Natural ingredient	Selenium	NP	Nrf2↑	Function:MMP↑, ATP↑ Mitochondrial dynamics:Drp1↓, Mff↓, Fis1↓, Opa1↑, Mfn1↑, Mfn2↑	Oxidative stress↓ Apoptosis↓	Wang et al. (2022b)
Natural ingredient	Urolithin A	NP	AMPK↑	Function:MMP↑ Mitophagy:LC3 II↑, P62↓	Apoptosis↓	Lin et al. (2020)
Natural ingredient	Mangiferin	NP	NF-κB↓	Function:MMP↑ Mitochondrial dynamics:Drp1↓, Opa1↑, TFAM↑	Inflammatory↓ ECM degradation↓ Oxidative stress↓ Apoptosis↓	Yu et al. (2021)
Natural ingredient	Polydatin	CEP	N/A	Function:mPTP↓, ΔΨm↑, ATP↑ Mitophagy:Parkin↑	Oxidative stress↓ Apoptosis↓	Kang et al. (2020b)
Hormone	Melatonin	NP	N/A	Function:ΔΨm↑, ATP↑ Mitophagy:Parkin↑, LC3 II/I↑, P62↓	ECM degradation↓ Apoptosis↑ ROS generation↓	Chen et al. (2019)
Hormone	Cortistatin	NP	NF-κB↓ AMPK/PGC-1α↑	Function:ΔΨm↑, ATP↑ Mitochondrial dynamics:Drp1↓, Opa1↑, Mfn1/2 ↑	ROS generation↓ NLRP3 inflammasome↓ Apoptosis↓	Zhao et al. (2020)
Gene Editing	circ-ERCC2	NP	miR-182-5p↓/SIRT1↑	Mitophagy:PINK1↓, Parkin↑, P62↓, LC3 II/I↑	ECM degradation↓ Senescence↓ Apoptosis↓	Xie et al. (2019)
Gene Editing	AV-Mfn2	NP	Mfn2↑	Function:ΔΨm↑ Mitophagy:PINK1↑, Parkin↑, LC3 II/I↑	ROS generation↓ Apoptosis↓	Chen et al. (2020b)
Gene Editing	LV-FOXO3	NP	FOXO3↑	Mitophagy:PINK1↑, Parkin↑, LC3 II/I↑, p62↓	ECM degradation↓ Apoptosis ↓	Wang et al. (2020b)
Gene Editing	PC-NDUFA4L2	NP	NDUFA4L2↑	Mitophagy:Parkin↓, LC3 II↓, p62↑	ROS generation↓ Excessive autophagy↓ Apoptosis↓	Wn et al. (2019)
Gene Editing	LV-NLRX1	NP	SLC39A7	Mitochondrial dynamics:OMA1 (ns), OPA1(ns), p-DNM1L (ns) Mitophagy:PINK1(ns), Parkin (ns), LC3 II/I↑	Senescence↓ ECM synthesis↑	Song et al. (2024)
Gene Editing	si-SPP1	NP	ITGα5/β1↓	Function:ΔΨm↑ Mitophagy:PINK1↑, Parkin↑, LC3 II/I↑, p62↓, ATG5↑, LAMP1↑	ECM synthesis↑ Apoptosis↓ Senescence↓ ROS generation↓	Gu et al. (2024)
Gene Editing	si-Drp1	AF	N/A	Function:MMP↑ Mitochondrial dynamics:Drp1↓	Apoptosis↓	Wu et al. (2021)
Targeted drugs	MitoQ	NP	Nrf2↑	Function:mPTP↓, ΔΨm↑ Mitochondrial dynamics:Drp1↓, Mff ↓, Fis1↓, Mfn1↑, Mfn2↑, Opa1↑ Mitophagy:PINK1↑, Parkin↑, LC3 II/I↑, p62↓	Oxidative stress↓ Apoptosis↓	Kang et al. (2020a)
Targeted drugs	Mito-TEMPO	CEP	N/A	Function:ΔΨm↑, ATP↑, mPTP↓ Mitophagy:Parkin↑	Oxidative stress↓ Apoptosis↓ ROS generation↓	Kang et al. (2020b)
Related protein	HSP70	NP	SIRT3↑	Function:MMP↑, ATP↑ Mitochondrial dynamics:Drp1↓, Mff↓, Fis1↓, Mfn1↑, Mfn2↑, Opa1↑	Oxidative stress↓ Apoptosis↓ ECM degradation↓	Hu et al. (2022)
Related protein	A20	NP	NLRP3 inflammasome↓	Function:ΔΨm↑ Mitochondrial dynamics:Drp1↓, Mfn1↑ Mitophagy:p62↓	Oxidative stress↓ Pyroptosis↓ Apoptosis↓ ROS generation↓	Peng et al. (2022)
Related protein	SIRT2	AF	PGC-1α↑	Mitophagy:Parkin↓, LC3 II↓	Oxidative stress↓ Apoptosis↓	Xu et al. (2019a)

Note: “↑” for upregulation (increased expression); “↓” for downregulation (decreased expression); “ns” represents no significant difference.

MMP, mitochondrial membrane potential; mPTP, mitochondrial permeability transition pore; oxLDL, oxidized Low-Density Lipoprotein; TBHP, tert-butyl hydroperoxide; AV, adenovirus; LV, lentivirus.

Beyond natural products, certain hormones have also been identified as possessing the potential to regulate mitophagy. For instance, Melatonin, an endogenous molecule released by the pineal gland, has been proven to effectively delay oxidative stress, inflammatory responses, and apoptosis in osteoarthritis models (Pei et al., 2009; Liu et al., 2013; Lim et al., 2012), while also enhancing mitophagy levels in various tissues such as the brain and liver (Lin et al., 2016; Kang et al., 2016). Chen et al. (Chen et al., 2019) found that Melatonin can promote mitophagy by upregulating Parkin protein expression and the LC3 II/I ratio, thereby improving oxidative stress-induced mitochondrial dysfunction and apoptosis, and exhibiting potential therapeutic effects on IVDD. Cortistatin, a cyclic neuropeptide, is an appealing therapeutic candidate in the treatment of degenerative and inflammatory diseases (Gonzalez-Rey et al., 2007; Duran-Prado et al., 2013; Gruber et al., 2014), including its role in mitigating TNF-α-induced chondrocyte inflammation to counteract articular cartilage degeneration in osteoarthritis (Zhao et al., 2019). Zhao et al. (Zhao et al., 2020) discovered that Cortistatin inhibits apoptosis by suppressing the NF-κB pathway and regulating mitochondrial dynamics, thereby reducing ROS accumulation and NLRP3 inflammasome activation. Specifically, in this study, Cortistatin, by activating the AMPK/PGC-1α pathway, upregulated the expression levels of fusion-related markers Opa1, Mfn1, and Mfn2, while simultaneously downregulating the expression of fission marker Drp1. Inhibition of proteins involved in mitochondrial fission also demonstrated a positive therapeutic effect on IVDD (Wu et al., 2021) (Table 2).

Targeted strategies aimed at mitochondrial function also constitute effective avenues for regulating mitophagy and ameliorating IVDD. Studies have confirmed that oxidative products are significantly increased in IVDD, and inhibiting the excessive production of ROS while promoting their clearance has been demonstrated to effectively delay the progression of IVDD [136–138] (Kang et al., 2020a; Suzuki et al., 2015). Among these, activating the Nrf2 antioxidant defense system emerges as a potent therapeutic strategy for IVDD. Nrf2, a crucial redox-sensitive transcription factor, regulates the antioxidant system by activating the expression of cytoprotective genes in response to oxidative stress (Xiang et al., 2022). For instance, antioxidants such as MitoQ and Mito-TEMPO significantly mitigate oxidative stress and mitochondrial dysfunction by activating the Nrf2 pathway and upregulating PINK1/Parkin-mediated mitophagy (Kang et al., 2020a; Kang et al., 2020b).

#### 4.2.2 Gene editing therapies

With the advancement of gene editing technology, an increasing number of studies have begun to explore their potential in regulating mitophagy and improving IVDD. For example, knocking down PINK1 expression using sh-PINK1 can affect the mitophagy process, leading to accelerated aging and increased ROS accumulation (Y et al., 2018). Additionally, circERCC2 promotes PINK1/Parkin-mediated mitophagy by downregulating miR-182-5p and upregulating SIRT1, thereby reducing NP cell apoptosis, senescence, and ECM degradation (Xie et al., 2019). Additionally, overexpression of genes such as Mfn2 and FOXO3 has also shown positive effects on mitophagy and IVDD treatment (Chen Y. et al., 2020; Wang et al., 2020b).

During the development of IVDD, various risk factors have been found to induce mitochondrial damage by inhibiting mitophagy, ultimately leading to RCD of disc cells. Therefore, enhancing mitophagy is considered a potential therapeutic approach for alleviating IVDD (Wang et al., 2020c; Lan et al., 2022). However, not all activation of mitophagy is positively correlated with halting the progression of IVDD. Excessive mitochondrial fission and autophagy can directly contribute to the occurrence and development of IVDD (Lin et al., 2023). Prolonged duration of mechanical loading has been reported to result in excessive removal of mitochondria by mitophagy, thus exacerbating NP cell senescence, and inhibiting mitophagy can have a positive effect (Huang et al., 2020). Further research has found that overexpression of the NDUFA4L2 gene exerts a positive therapeutic effect on IVDD by inhibiting excessive mitophagy induced through the Parkin-dependent pathway (Wn et al., 2019). NLRX1, as the only Nod-like receptor located in mitochondria, plays a crucial role in sensing mitochondrial damage and regulating mitochondrial function (Zhang et al., 2019; Killackey et al., 2022; Killackey et al., 2023). Song et al. (Song et al., 2024) revealed that mitophagy is activated in both NLRX1-overexpressing and NLRX1-deficient NP cells exposed to oxidative stress. However, distinct biological outcomes were observed. When NLRX1 was overexpressed, pharmacological intervention targeting the NLRX1-SLC39A7 pathway showed great potential for promoting disc regeneration. Conversely, NLRX1 deficiency promoted PINK1/Parkin-mediated mitophagy, inducing excessive mitophagy and accelerating the progression of IVDD. Therefore, from a mechanistic perspective, the zinc transporter SLC39A7, a novel NLRX1-interacting protein, has been identified and proven to regulate mitochondrial dynamics and promote beneficial and synchronized mitophagy (Song et al., 2024) (Table 2).

#### 4.2.3 Protein-based therapies

Proteins such as HSP70 also mitigate oxidative stress and apoptosis by modulating mitochondrial dynamics and the expression of autophagy-related proteins (Hu et al., 2022). Furthermore, proteins including A20 and SIRT2 have been identified as possessing potential to regulate mitophagy (Xu W-N. et al., 2019; Peng et al., 2022). In the study by Peng et al. (Peng et al., 2022), following LPS treatment, the mitochondrial fission protein Drp1 translocated from the cytoplasm to mitochondria, while the expression of Mfn1 significantly decreased. A20 mitigated the LPS-induced changes and promoted the normalization of mitochondrial morphology. A20 significantly reduced the NLRP3 aggregation around mitochondria induced by LPS. Analysis revealed that A20 protected NP cells from LPS-induced mPTP collapse and massive ROS production. These findings suggest that A20 may exert protective effects by facilitating the elimination of ROS through mitophagy.

### 4.3 Mitochondrial dynamics

On the other hand, mitochondria are highly dynamic organelles undergoing continuous fission and fusion, a process termed mitochondrial dynamics (Westermann, 2010a). When cells undergo metabolic or environmental stress, the quantity and quality of mitochondria are regulated through continuous processes of fusion and fission (Pernas and Scorrano, 2016; Kraus and Ryan, 2017). Fusion aids in stress alleviation by mixing the contents of partially damaged mitochondria as a form of complementation. Fission is necessary for the generation of new mitochondria, but it also contributes to quality control by enabling the removal of damaged mitochondria and can facilitate apoptosis under high levels of cellular stress (Youle and van der Bliek, 2012). Mitochondrial fusion and fission are crucial for a wide range of cellular functions, including energy metabolism, development, aging, and cell death. Mitochondrial fusion and fission are crucial for a great variety of cellular functions, including energy metabolism, development, aging and cell death. The core mechanisms involved have been identified and analyzed in diverse model organisms (Westermann, 2010b). A delicate balance in mitochondrial dynamics is conducive to maintaining a healthy mitochondrial pool (Lee and Yoon, 2016). Disruption of this balance is associated with various human diseases, including cancer, type 2 diabetes, and osteoarthritis (Rovira-Llopis et al., 2017; Srinivasan et al., 2017; Yao et al., 2019). Mitophagy and mitochondrial dynamics are interrelated but distinct processes. During the process of mitochondrial fission, damaged daughter mitochondria are first segregated and subsequently targeted for elimination by lysosomes, thereby preventing their reintegration into the pool of active and healthy mitochondria through fusion (Kang et al., 2020a). Maintaining a healthy mitochondrial pool is crucial for disc cells in both physiological adaptation and pathological responses to external stimuli. Therefore, in addition to direct interventions targeting mitophagy, appropriate mitochondrial quality control also merits investigation.

## 5 Summary and prospects

IIVDD is closely associated with a range of spinal disorders, including lumbar disc herniation, spinal canal stenosis, degenerative spondylolisthesis, and scoliosis, which impose significant economic burdens on both individuals and society (Hartvigsen et al., 2018). During the progression of IVDD, various modes of RCD exist within the intervertebral disc, such as apoptosis, pyroptosis, senescence, and autophagy-dependent cell death, which can occur independently or in combination (Yang et al., 2022). To date, effective therapeutic strategies for IVDD are still under continuous exploration.

By Delving into the mechanisms underlying the role of mitochondrial function in IVDD, the modulation of mitophagy has emerged as one of the important directions for improving therapeutic strategies for IVDD. Various interventions, including natural products, hormones, targeted compounds, gene editing technologies, and related proteins, have demonstrated positive effects on mitophagy, providing new directions and strategies for the treatment of IVDD. However, enhancing mitophagy is not always a favorable approach for treating IVDD, and treatment decisions should be based on a clear understanding of the molecular background. Therefore, whether mitophagy is beneficial or harmful to health depends on cellular and microenvironmental factors (Zhou et al., 2019). Meanwhile, both mitophagy and mitochondrial dynamics are key mechanisms for maintaining mitochondrial homeostasis (K et al., 2018).

Current research still faces numerous challenges and unknown areas, such as the interactions between different mechanisms and the long-term safety and efficacy of intervention methods. Therefore, future studies need to explore the specific mechanisms of these strategies in greater depth and validate and optimize them in clinical practice, aiming to provide more effective and safe treatment options for patients.
